# Pitfalls and Pearls in Delusional Parasitosis

**DOI:** 10.5811/cpcem.2019.8.44619

**Published:** 2019-10-14

**Authors:** Allen Gold, Zhanna Roit, Ingrid Llovera

**Affiliations:** Northwell Health, Emergency Department, Manhasset, New York

## Abstract

Delusional parasitosis is an uncommon psychiatric disorder that manifests as having parasitic delusions. Due to its rarity, delusional parasitosis is a challenging and costly diagnosis of exclusion and proves difficult to manage for many providers. Although this syndrome is frequently discussed in psychiatric and dermatology reports, it is not commonly described in emergency medicine (EM) literature. As a result, best practices for workup and treatment remain unclear from an EM perspective. Patients typically return multiple times for medical evaluation and exhaust numerous resources. In this case report we review the appropriate steps for initial evaluation of patients with suspected delusional parasitosis, differential diagnoses, and increase awareness for prudent treatment strategies.

## INTRODUCTION

We present a patient with delusional parasitosis who had a typical, although turbulent, medical workup in the emergency department (ED). Alternative organic causes to explain his behavioral changes were successfully ruled out. However, following an exhaustive compilation of tests and frustrating return visits, the patient still did not receive the proper treatment and was eventually lost to follow-up.

## CASE REPORT

A 48-year-old male with a past medical history of cystic acne, but otherwise insignificant medication and social history, presented with concern for a parasite on his face. He reported that on the previous night he thought a parasite had crawled out of a healed cystic lesion on his right cheek. He presented a jar with a bloody napkin and paper, stating that he had scraped the parasite from his face with a scalpel and brought it with him to the ED. His physical exam was notable for diffuse excoriations and scaly patches, a 3 × 4 centimeter (cm) abrasion on his right cheek and mild anxiety. Otherwise his vital signs and physical exam were unremarkable.

No obvious parasites were appreciated on examination of his skin or in the jar. Basic blood tests including a complete blood count (CBC) and basic metabolic panel were within normal limits. A urine toxicology test was evaluated and reported negative. During his stay, the patient called a provider to the bedside to evaluate his thumb for a parasite actively crawling out of his skin. The provider documented that no obvious insects or wounds were apparent at that time. Delusional parasitosis was suspected and psychiatry was consulted, but the patient eloped from the ED prior to being evaluated.

The patient returned to the ED two days later with a complaint of parasitic infection. This time he described seeing white maggots crawling from his skin and presented us with a jar with a flaky material. The patient presented to the outpatient infectious disease (ID) clinic and was directed to come to the ED to obtain a formal ID consult. Although there was low clinical suspicion for an infectious component, ID was consulted for the presumed benefit of reassurance from a consultation service. The flakes presented by the patient were analyzed for ova and parasites and reported to the patient as negative. He was then evaluated by psychiatry who reported that his presentation was consistent with a delusional parasitosis but did not recommend inpatient hospitalization given that he did not appear to be at risk for harm to himself or others. He eloped a second time prior to receiving discharge papers.

The patient returned for a third visit to the ED with similar complaints. His workup included basic labs and a non-contrast head computed tomography (CT), which were all unremarkable. He was instructed to follow up with psychiatry as an outpatient.

## DISCUSSION

This case represents a patient with delusional parasitosis, also known as Ekbom syndrome, psychogenic parasitosis, dermatophobia, chronic tactile hallucinosis, parasitophobia, and cocaine bugs. This disease is rare, occurring at an estimated incidence of 1.9 cases/100,000 person-years. The average demographic affected by delusional parasitosis is female in her late 50s with almost a 3:1 female to male incidence.^1^ Delusional parasitosis is described as a condition where a patient has a fixed false belief that he or she is infected by parasitic organisms and cannot be persuaded otherwise.^2^ These patients typically function normally in their daily lives outside of this focused delusion.

Primary delusional parasitosis occurs when the delusion is the only manifestation. Secondary delusional parasitosis occurs when the delusion is merely a symptom that occurs in the setting of another psychiatric disorder, medical illness, or substance abuse.^3^,^4^ Patients with delusional parasitosis typically present to the ED seeking medical and dermatologic care. Pruritus is typically described, occurring over the course of months. Patients often times present “parasitic” specimens, which are actually scabs, cloth fibers, and other materials. The physical exam is typically notable for multiple ulcerations, excoriations, and scars secondary to intense scratching with fingernails, knives, or pins.^5^

Although primary delusional parasitosis is a relatively benign disease, the role of an emergency physician (EP) is to rule out other secondary diseases that manifest as parasitic delusions. A high level of suspicion for a secondary cause should be maintained in younger patients. Common disease culprits for secondary delusional parasitosis are head trauma, dementia, cerebrovascular disease, thyroid dysfunction, nutritional deficiencies (eg, niacin/cobalamin [B12]/folate), substance abuse, encephalitis, and actual parasitic infestations.^5^

Patients should be queried about recent travel history and exposure to known infected individuals with scabies or bed bugs. An objective ED workup that may help elucidate whether an underlying disease process is occurring includes a CBC (for eosinophilia), thyroid stimulating hormone, B12, folate, glucose, urea, liver function tests, urine toxicology, syphilis screen, human immunodeficiency virus testing, and head CT based on the history and physical exam. Lastly, some prescription and illicit drugs that have been associated with delusional parasitosis include ciprofloxacin, corticosteroids, topiramate, ketoconazole, and chronic alcohol (and withdrawal), cocaine and amphetamine use.^5^,^6^,^7^,^8^

Delusional parasitosis is a difficult syndrome to treat given patient noncompliance.^9^ Patients typically deny that they have a delusional disorder and will seek multiple doctors and specialists to validate their symptoms.^10^ Patients are often lost to follow-up since they commonly mistake their doctor’s recommendations as a sign of incompetence or apathy. Therefore, outpatient providers are challenged with the difficult task of establishing a therapeutic and trusting relationship with the patient so that they may eventually accept recommendations and become compliant with treatment.

**CPC-EM Capsule**

What do we already know about this clinical entity?

*Delusional parasitosis is a difficult diagnosis of exlcusion to make in the emergency department. Treatment and close follow-up are often unsuccessful given the vulnerable patient population*.

What makes this presentation of disease reportable?

*This presentation of delusional parasitosis is fairly common but outlines common pitfalls in diagnostic work-up and treatment of which many providers are unaware*.

What is the major learning point?

*An important treatment approach for delusional parasitosis is acknowledgement of a real medical problem, initiation of antispychotic therapy, reassurance, and proper followup with a trusted physician*.

How might this improve emergency medicine practice?

*A more tactical treatment approach may reduce emergency department repeat visits and wasted resources*.

A systematic review of multiple case series and observational studies showed a 60–100% efficacy rate with antipsychotic medications such as pimozide, olanzapine, or risperidone.^11^,^12^ Randomized controlled trials are difficult to conduct with this patient population, but some retrospective reviews note no difference between first- and second-generation antipsychotics in treatment success for delusional parasitosis.^12^ A second-generation antipsychotic may be the more prudent treatment option given the lower rate of extrapyramidal side effects.^13^,^14^

EPs can play a role in treatment by reassuring these patients that there is no acute illness going on. Reassurance should be supported by negative laboratory and imaging studies. Furthermore, EPs should delicately acknowledge the patient’s concern, being careful not to disregard them in a cavalier manner, and recommend close follow-up with a physician with whom the patient can develop a trusting relationship.

## CONCLUSION

EPs have the difficult task of discerning between psychiatric and organic causes of mental illness. Even when successfully identified, many psychiatric diseases are still challenging to treat due to unfamiliarity with the disorder, vulnerable patient population, and tendency for return visits. Delusional parasitosis represents one rare psychiatric illness that emergency providers may become more familiar with and thus develop better recommendations to ensure treatment compliance and close follow-up. Here we have outlined the appropriate history, physical exam, important differential diagnoses, and treatments that emergency physicians should be familiar with to manage delusional parasitosis.
